# Bacterial Etiologies of Five Core Syndromes: Laboratory-Based Syndromic Surveillance Conducted in Guangxi, China

**DOI:** 10.1371/journal.pone.0110876

**Published:** 2014-10-31

**Authors:** Baiqing Dong, Dabin Liang, Mei Lin, Mingliu Wang, Jun Zeng, Hezhuang Liao, Lingyun Zhou, Jun Huang, Xiaolin Wei, Guanyang Zou, Huaiqi Jing

**Affiliations:** 1 Department of Emergency Response, Guagnxi Zhuang Autonomous Region Health Bureau, Nanning, Guangxi, China; 2 Divsion of Bacterial Infectious Disease Control, Guagnxi Zhuang Autonomous Region Center for Disease Prevention and Control, Nanning, Guangxi, China; 3 School of Public Health and Primary Care, Chinese University of Hong Kong, Hong Kong, China; 4 COMDIS Health Services Delivery Research Consortium, China Program, Nuffield Centre for International Health and Development, University of Leeds, Shenzhen, China; 5 National Institute for Communicable Disease Control and Prevention, Chinese Center for Disease Control and Prevention, Beijing, China and State Key Laboratory for Infectious Disease Prevention and Control, Collaborative Innovation Center for Diagnosis and Treatment of Infectious Diseases, Beijing, China; University of Texas Medical Branch, United States of America

## Abstract

**Background:**

Under the existing national surveillance system in China for selected infectious diseases, bacterial cultures are performed for only a small percentage of reported cases. We set up a laboratory-based syndromic surveillance system to elucidate bacterial etiologic spectrum and detect infection by rare etiologies (or serogroups) for five core syndromes in the given study area.

**Methods:**

Patients presenting with one of five core syndromes at nine sentinel hospitals in Guagnxi, China were evaluated using laboratory-based syndrome surveillance to elucidate bacterial etiologies. We collected respiratory and stool specimens, as well as CSF, blood and other related samples for bacterial cultures and pulse field gel electrophoresis (PFGE) assays.

**Results:**

From February 2009 to December 2011, 2,964 patients were enrolled in the study. Etiologies were identified in 320 (10.08%) patients. *Streptococcus pneumonia* (37 strains, 24.18%), *Klebsiella pneumonia* (34, 22.22%), *Pseudomonas aeruginosa* (19, 12.42%) and *Haemophilus influenza* (18, 11.76%) were the most frequent pathogens for fever and respiratory syndrome, while *Salmonella* (77, 81.05%) was most often seen in diarrhea syndrome cases. *Salmonella paratyphi A* (38, 86.36%) occurred in fever and rash syndrome, with *Cryptococcus neoformans* (20, 35.09%), *Streptococcus pneumonia* (5, 8.77%), *Klebsiella pneumonia* (5, 8.77%),*streptococcus suis* (3, 5.26%) and *Neisseria meningitides group B* (2, 3.51%) being the most frequently detected in encephalitis-meningitis syndrome. To date no pathogen was isolated from the specimens from fever and hemorrhage patients.

**Conclusions:**

In addition to common bacterial pathogens, opportunistic pathogens and fungal infections require more attention. Our study contributes to the strengthening of the existing national surveillance system and provides references for other regions that are similar to the study area.

## Introduction

Since 2003, a real-time web-based disease surveillance system was developed to monitor 39 notifiable diseases in China. This system was based on the existing reporting system for selected infectious diseases established in the 1950s [1]. This system consists of a 5-level reporting network that ranges from township, county, prefecture, province and finally to the national level. Infectious diseases can be reported to the Central Data Bank in China Center for Disease Control and Prevention (or CDC) whenever they are detected at hospitals in any of these five levels. Besides the above-mentioned 39 known diseases, pneumonia of unknown cause is required to be reported through the system. This surveillance system makes timely nation-wide disease reporting and early outbreak detection possible.

Nevertheless, the national surveillance system does have limitations. Under this system, reported cases of bacterial infection were determined mainly by clinical features without accompanying laboratory confirmation, since bacterial culture is not routinely performed for suspected infection cases in hospitals. As a result, defining an overall picture of the etiological spectrum is difficult. In addition, the national system merely includes commonly known diseases and pneumonia of unknown cause, and does not consider rare or emerging diseases that present with other syndromes.

Besides the national infectious disease surveillance system, more specific surveillance activities were conducted to explore the prevalence and etiology of bacterial infectious diseases [2], [3]. But these previous surveillance efforts were aimed at a single syndrome or pathogen.

Currently, limited data exist for the integrated surveillance of various syndrome etiologies in China. In 2011 a systematic surveillance network was set up in Guangxi, China to monitor mainly fever and respiratory, diarrhea, fever and rash, fever and hemorrhage and encephalitis-meningitis syndromes. With signs and syndromes as a starting point, clinical information and specimens can be collected at the early stages of these diseases. Use of syndromic surveillance can determine the etiological spectrum of infectious diseases through multiple-etiology testing on such specimens. This paper aims to explore bacterial etiology spectra for five core syndromes: fever and respiratory, diarrhea, fever and rash, fever and hemorrhage and encephalitis-meningitis under a laboratory-based syndromic surveillance system. Results from our study will contribute to the strengthening of the existing national surveillance system and provide references for other settings that are similar to the study area on strategy development for vaccine introduction and infectious disease surveillance, as well as on bacterial etiology study.

## Materials and Methods

### Set-up of the surveillance network

The Guangxi Zhuang Autonomous Region (or Guangxi) is one of the provincial administrative regions in China, and is located in the southwestern region of the nation. Guangxi had a population of 48,160,001 at the end of 2009 and is divided into 14 areas called “prefecture-level-city” (or prefecture). Each prefecture is composed of a number of counties (including counties, districts and county-level cities). There are 119 counties in Guangxi.

A total of 12 counties/districts in 4 prefectures located in southern, central or northern Guangxi were selected as surveillance sites. There were 6 districts in the Nanning prefecture and 1 county in the Beihai prefecture located in southern Guangxi. Meanwhile, central Guangxi was represented by 4 districts/counties of the Guiguang prefecture and in northern Guangxi 1 county of the Guilin Prefecture was included. The health facilities in these selected sites were experienced in bacterial disease surveillance and capable of conducting such systematic surveillance.

**Figure 1:**
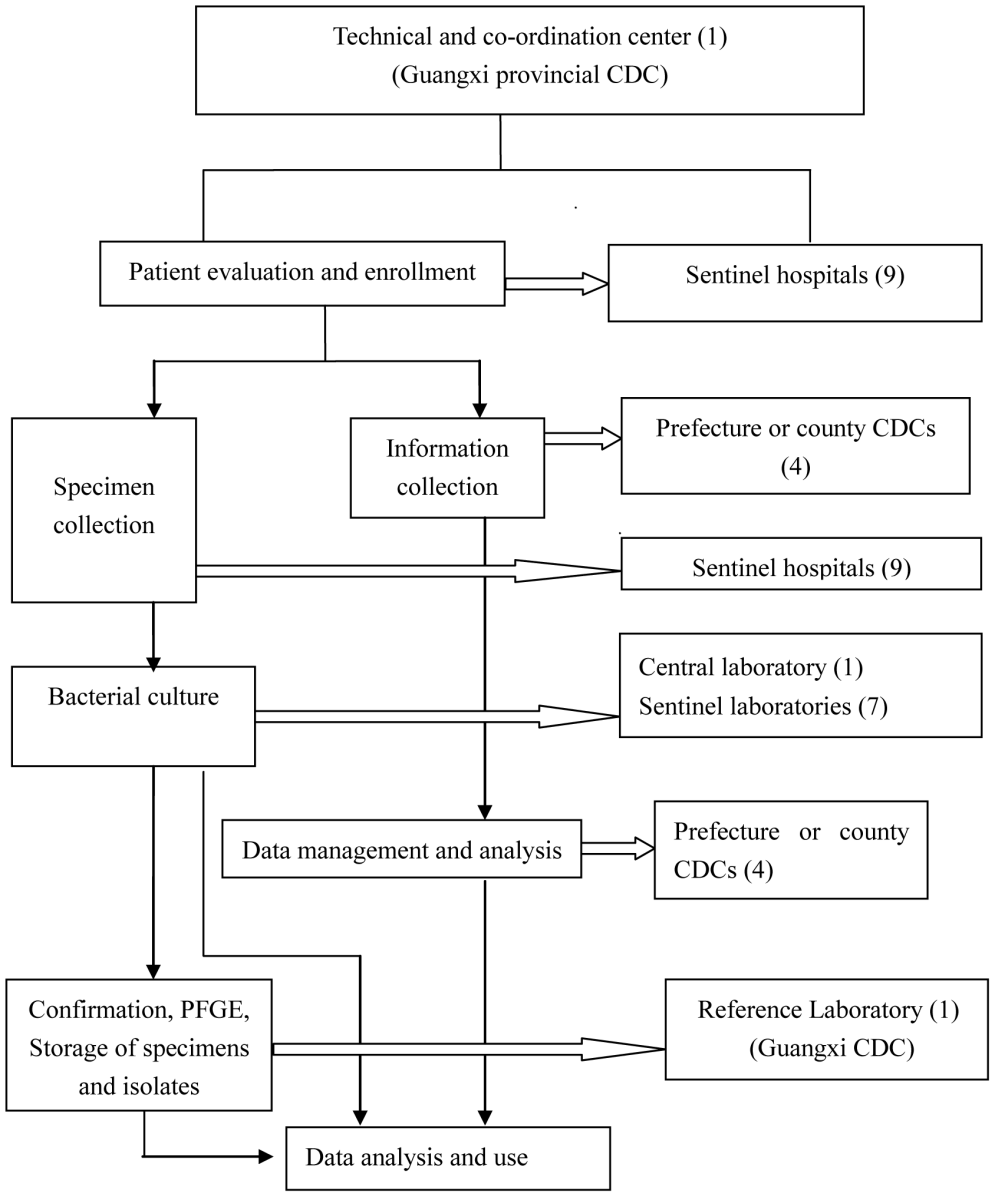
Surveillance network for the five core syndromes.

The surveillance network consisted of the provincial CDC, 1 prefecture CDC, 3 County CDCs, 9 sentinel hospitals (2 provincial level, 4 prefecture-level and 3 county-level), 1 central laboratory (provincial level) and 7 surveillance laboratories (affiliated with the sentinel hospitals) (Figure 1). The physicians at the sentinel hospitals evaluated cases and enrolled eligible cases that met the case definitions into the surveillance network. Relevant information and clinical specimens from the enrolled cases were collected. The CDC health workers regularly visited the sentinel hospitals to search for cases meeting the surveillance definition according to the standard operational procedures of this surveillance network.

The surveillance for encephalitis-meningitis syndrome covered the period from February 2009 to December 2011. For the remaining 4 syndromes, the study period was from June 2010 to December 2011.

### Case Definitions

Patients presenting to the sentinel hospitals who met any of the following definitions [4] were enrolled in the surveillance system:

- Fever and respiratory syndrome: including upper respiratory infection (URI) and pneumonia. URI was characterized as fever with axillary temperature ≥37.3°C, weakness, headache, cough, sore throat or muscle pain. Pneumonia cases with inflammation in the end-expiratory airway, pulmonary alveolus and pulmonary interstitium that was characterized as fever, cough and accompanied by polypnea, tachypnea with positive chest radiological findings were also included.
- Diarrhea syndrome: passage of three or more loose, liquid, bloody, purulent or mucoid stools in any 24-hour period.
- Fever and rash syndrome: fever with axillary temperature ≥37.3°C lasting more than 1 day and rash presenting on the skin. Suspected cases of typhoid and paratyphoid fever in endemic areas were also enrolled.
- Fever and hemorrhage syndrome: fever with axillary temperature ≥37.3°C and hemorrhage in the skin, mucosa or other parts of the body.
- Encephalitis-meningitis syndrome: Fever, headache, vomiting, accompanied by altered consciousness or meningeal irritation, including various central nervous system symptoms.

### Specimen Collection

Appropriate clinical specimens were collected from patients enrolled with the five core syndromes:

- Specimens collected for fever and respiratory syndrome included a blood specimen, one throat swab and a sputum specimen. A pleural effusion specimen,a bronchoalveolar lavage fluid specimen and a nasopharyngeal aspirate specimen were collected as needed.
- A stool specimen was collected for each eligible case with diarrhea syndrome.
- Specimens collected for fever and rash syndrome included a blood specimen and one throat swab. A stool specimen and pus specimen from the skin were collected as needed.
- A blood specimen was collected for each eligible case with fever and hemorrhage syndrome. An exudate specimen from the skin and a cerebrospinal fluid specimen (CSF) were collected as needed.
- A blood and CSF specimen were collected for cases with encephalitis-meningitis syndrome.

### Data collection and Analysis

Standardized case information forms were used to collect information on identification, epidemiology, clinical features, laboratory analysis and clinical specimens of enrollees with the five core syndromes.

To measure the prevalence of laboratory-confirmed bacterial infection, the percentage of positive cultures was calculated by specimens and syndromes. To characterize the bacterial spectrum, the proportions were calculated for each key etiology isolated from the five syndromes. To analyze the epidemiologic link between cases with identical pulsed field gel electrophoresis (PFGE) patterns, we compared the information for demography, clustering, addresses, sentinel hospitals and date of specimen collection. Data were analyzed using SPSS 13.0. A chi-square test was performed to test for statistical differences in bacterial culture results between genders, locations and ages. A *P* value <0.05 was considered statistically significant.

### Laboratory Testing

Bacterial culturing of clinical specimens was performed according to routine operational procedures [5] (Table 1). Bacterial identification was done using the API biochemical identification system (bioMérieux, Marcy-l'Etoile, France) according to the manufacturer's guidelines.

**Table 1:** Methods of bacterial culture for five core syndromes.

Specimen	Enrichment			Culture/subculture			Syndrome	Bacterial pathogens detected
	Media	Conditions	Length of Incubation	Media*	Conditions	Length of Incubation		
Blood	Aerobic Blood culture bottle	5%CO_2_,35°C	Day 1,2,4,7	Blood and chocolate agar	5%CO_2_,35°C	for up to 48 hours	Encephalitis-meningitis	*N.meningitidis, H.influenzae, Staphylococci, Streptococci, E. coli, Cryptococcus, others (Corynebacterium, K.pneumonia, M.luteus, H.alvei, Salmonella, S.maltophilia)*
	Aerobic Blood culture bottle	5%CO_2_,35°C	Day 1,2,4,7	Blood and chocolate agar	5%CO_2_,35°C	for up to 48 hours	Fever and rash	*S.typhi, S.paratyphi A*
	Aerobic Blood culture bottle	5%CO_2_,35°C	Day 1,2,4,7	Blood and chocolate agar	5%CO_2_,35°C	for up to 48 hours	Fever and hemorrhage	*S.suis*
	Aerobic Blood culture bottle	5%CO_2_,35°C	Day 1,2,4,7	Blood and chocolate agar	5%CO_2_,35°C	for up to 48 hours	Fever and respiratory	*Streptococci, Staphylococci, K.pneumoniae, P.aeruginosa, H.influenzae, others ( Candida, E.coli, Acinetobacter, B.cepacia, S.maltophilia, S.marcescens, C.meningosepticum, O.anthropi, P.fluorescens, E.aerogenes, E.cloacae)*
Sputum	Not needed	/	/	Chocolate, MacConkey and blood agar	5%CO2,35°C	18–24 h	Fever and respiratory	*Streptococci, Staphylococci, K.pneumoniae, P.aeruginosa, H.influenzae, others ( Candida, E.coli, Acinetobacter, B.cepacia, S.maltophilia, S.marcescens, C.meningosepticum, O.anthropi, P.fluorescens, E.aerogenes, E.cloacae)*
Nasopharyngeal aspirate	Not needed	/	/	Chocolate, MacConkey and blood agar	5%CO2,35°C	for up to 48 hours	Fever and respiratory	*Streptococci, Staphylococci, K.pneumoniae, P.aeruginosa, H.influenzae, others ( Candida, E.coli, Acinetobacter, B.cepacia, S.maltophilia, S.marcescens, C.meningosepticum, O.anthropi, P.fluorescens, E.aerogenes, E.cloacae)*
Bronchoalveolar lavage fluid	Not needed	/	/	Chocolate, MacConkey and blood agar	5%CO2,35°C	for up to 48 hours	Fever and respiratory	*Streptococci, Staphylococci, K.pneumoniae, P.aeruginosa, H.influenzae, others ( Candida, E.coli, Acinetobacter, B.cepacia, S.maltophilia, S.marcescens, C.eningosepticum, O.anthropi, P.fluorescens, E.aerogenes, E.cloacae)*
Pleural effusion	Not needed	/	/	Blood and chocolate gar	5%CO2,35°C	for up to 48 hours	Fever and respiratory	*Streptococci, Staphylococci, K.pneumoniae, P.aeruginosa, H.influenzae, others ( Candida, E.coli, Acinetobacter, B.cepacia, S.maltophilia, S.marcescens, C.meningosepticum, O.anthropi, P.fluorescens, E.aerogenes, E.cloacae)*
Throat swab	Not needed	/	/	Chocolate, MacConkey and blood agar	5%CO2,35°C	18–24 h	Fever and respiratory	*Streptococci, Staphylococci, K.pneumoniae, P.aeruginosa, H.influenzae, others ( Candida, E.coli, Acinetobacter, B.cepacia, S.maltophilia, S.marcescens, C.meningosepticum, O.anthropi, P.fluorescens, E.aerogenes, E.cloacae)*
	Not needed	/	/	Chocolate, MacConkey and blood agar	5%CO2,35°C	18–24 h	Fever and rash	*Streptococcus*
Stool	Not needed	/	/	MacConkey, SSagar,XLD agar	35°C	18–24 h	Diarrhea	*E.coli, Shigella, Salmonella*
	APW	35°C	18–24 h	MacConkey agar	35°C	18–24 h	Diarrhea	*P.shigelloides*
	APW	35°C	18–24 h	Ampicillin MacConkey agar	35°C	18–24 h	Diarrhea	*Aeromonas*
	APW	35°C	18–24 h	TCBS	35°C	18–24 h	Diarrhea	*Pathogenic Vibrios*
	SBG	35°C	18–24 h	Chromogenic Salmonella agar	35°C	18–24 h	Diarrhea	*Salmonella*
	PBS	4°C	10 days	CIN	25°C	3 days	Diarrhea	*Yersinia*
	Not needed	/	/	CCDA	Microaerophilic,42°C	3–5 days	Diarrhea	*Campylobacter*
	Not needed	/	/	Blood agar	35°C	18–24 h	Fever and rash	*S.typhi, S.paratyphi A, others(S.maltophilia)*
Cerebrospinal fluid	Not needed	/	/	Chocolate, MacConkey and blood agar	5%CO2,35°C	for up to 48 hours	Encephalitis-meningitis	*N.meningitidis, H.influenzae, Staphylococci, Streptococci, E.coli, Others(K.pneumoniae, P.aeruginosa, Salmonella)*
	Not needed	/	/	Chocolate, MacConkey and blood agar	5%CO2,35°C	for up to 48 hours	Fever and hemorrhage	*S.suis*
Pus	Not needed	/	/	Blood agar	5%CO2,35°C	for up to 48 hours	Fever and rash	*Streptococcus, others (S.aureus)*
Exudate	Not needed	/	/	Hottinger's Agar	28°C	for up to 48 hours	Fever and hemorrhage	*Y.pestis*

*APW Alkaline Peptone Water.

SBG Selenite brilliant green sulfa enrichment broth.

PBS Phosphate buffered saline.

CIN Ccefsulodin-irgasan-novobiocin agar.

SS Salmonella-Shigella agar.

XLD Xylose lysine desoxycholate agar.

TCBS Thiosulfate citrate bile salts sucrose agar.

CCDA Charcoal cefoperazone deoxycholate agar.

Opportunistic pathogens were considered clinically significant if the organisms grew dominantly on culture media or the same organisms were detected from more than 2 cultures or from different types of samples. And the results were reported when supported by both laboratory data and clinical data.

PFGE was performed with the *Not*I restriction enzyme (Invitrogen) according to the standard operating procedure for Pulsenet (Pulsenet International) PFGE of *Escherichia coli* O157: H7, *E. coli* NON-0157 (STEC), *Salmonella* serotypes, *Shigella sonnei* and *Shigella flexner*. Plugs were submitted to electrophoresis using CHEF Mapper equipment (BioRad). The Lambda PFGE marker (Biolabs) was used in each assay. Images of the pulsetypes were uploaded and analyzed using BioNumerics software, Version 5.1 (Applied Maths).

### Quality Assurance and Control

Standard operational procedures (SOPs) for the surveillance were formulated and SOP training was given to standardize the surveillance activities. Health staff at the CDCs visited the sentinel hospitals and sentinel laboratories every other week to assure the quality of case evaluation, data collection, specimen collection and testing. The completed case information forms were reviewed by specific data managers for completeness and logic errors. Proficiency tests were performed in sentinel laboratories at irregular intervals.

### Ethical Considerations

The study protocol was reviewed and approved by the Ethics Committee of Guangxi Zhuang Autonomous Region in January 2009. Signed informed consent was obtained from all study participants. For patients <18 years of age, a written consent form was signed by a parent or legal guardian.

## Results

### Demographics of Enrolled Patients

A total of 2,964 enrollees were evaluated from February 2009 to December 2011. Of these, 1,832 (61.81%) were male and 1,132 (38.19%) were female; 1,421 (47.94%) lived in urban areas and 1,543 (52.06%) in rural areas. The median age of the patients enrolled was 4 years (range, 0 to 119 years). Of these, 905 (30.53%) were patients with fever and respiratory syndrome, 939 (31.68%) with diarrhea syndromes, 319 (10.76%) with fever and rash syndrome, 111 (3.74%) with fever and hemorrhage syndrome and 690 (23.28%) with encephalitis/meningitis syndrome.

### Positive Culture by Specimen Type

Of 5,907 specimens collected from 2,964 enrollees, 349 (5.91%) produced positive cultures for bacterial etiologies. For fever and respiratory syndrome, pathogens were isolated from 14.71% of the sputum specimens. For diarrhea syndrome, 10.08% of the stool specimens were culture positive. For fever and rash syndrome, 10.55% of blood specimens were culture positive. For encephalitis-meningitis syndrome, pathogens were isolated from 3.60% of blood specimens and 5.19% of CSF specimens. No pathogen was isolated for fever and hemorrhage syndrome (Table 2).

**Table 2:** Positive Cultures by specimen type for five core syndromes.

Syndrome	Specimen	No. tested	No. positive	% Positive
Fever and Respiratory	Sputum	700	103	14.71
	Throat/nasal swab	832	22	2.64
	Blood	1342	26	1.94
	Pleural effusion	9	1	11.11
	Bronchoalveolar lavage fluid	14	1	7.14
	Nasopharyngeal aspirate	11	0	0.00
Diarrhea	Stool	942	95	10.08
Fever and rash	Blood	379	40	10.55
	Stool	7	1	14.29
	Throat swab	239	1	0.42
	Pus	5	2	40.00
Fever and hemorrhage	Blood	114	0	0.00
	Exudate	2	0	0.00
Encephalitis-meningitis	Blood	694	25	3.60
	CSF	617	32	5.19
Total		5907	349	5.91

### Culture Positive by five Core Syndromes

Bacterial pathogens were isolated from 320 (10.80%) cases out of 2,964 enrollees with one of the five core syndromes.

Pathogens were isolated from 140 (15.47%) out of 905 patients with fever and respiratory syndrome. Significantly higher positive rates were observed in patients living in rural areas (22.38%) compared to those in urban areas (11.05%) (P = 0.000), in males (17.29%) than in females (12.15%) (P = 0.041), and in patients aged 0∼10 years (19.11%) than in patients >10 years (9.57%) (P = 0.000).

Pathogens were isolated from 93 (9.90%) out of 939 patients with diarrhea syndrome. No statistically significant differences in positive rates were observed between patients living in rural areas (9.22%) and urban areas (10.22%) (P = 0.643), between males (9.46%) and females (10.60%) (P = 0.568), between patients aged 0∼10 years (8.54%) and >10 years of age (10.59%) (P = 0.320).

Pathogens were isolated from 42 (13.17%) out of 319 patients with fever and rash syndrome. Significantly higher positive rates were observed in patients living in urban areas (33.94%) than in rural areas (2.38%) (P = 0.000), in females (18.38%) than males (9.29%) (P = 0.018), and in patents >10 years of age (17.65%) than those aged 0∼10 years (3.06%) (P = 0.000).

For encephalitis-meningitis syndrome pathogens were isolated from 45 (6.52%) out of 690 patients. No significant difference in positive rates was observed between patients living in rural areas (5.96%) and urban areas (9.71%) (P = 0.603), males (6.90%) and females (5.88%) (P = 0.156). Higher positive rates were found in children aged 0∼10 years (4.04%) than patients >10 years of age (9.86%) (P = 0.002) (Table 3).

**Table 3:** Culture positive by five core syndromes.

	Fever and respiratory		Diarrhea		Fever and rash		Encephalitis-meningitis	
	Cases tested	No.positive (%)	Cases tested	No.positive (%)	Cases tested	No.positive (%)	Cases tested	No.positive (%)
Location								
urban	552	61(11.05)	646	66(10.22)	109	37(33.94)	103	10(9.71)
rural	353	79(22.38)	293	27(9.22)	210	5(2.38)	587	35(5.96)
X^2^		21.132		0.227		62.53		2.017
P		0		0.634		0		0.156
Gender								
Male	584	101(17.29)	571	54(9.46)	183	17(9.29)	435	30(6.90)
Female	321	39(12.15)	368	39(10.60)	136	25(10.38)	255	15(5.88)
X^2^		4.193		0.326		5.642		0.271
P		0.041		0.568		0.018		0.603
Age								
0–10 years	560	107(19.11)	623	66(10.59)	98	3(3.06)	396	16(4.04)
>10 years	345	33(9.57)	316	27(8.54)	221	39(17.65)	294	29(9.86)
X^2^		14.864		0.987		12.634		9.386
P		0		0.32		0		0.002
Total	905	140(15.47)	939	93(9.90)	319	42(13.17)	690	45(6.52)

*To date no bacterial pathogen has been detected from specimens of fever and hemorrhage syndrome.

### Etiologic Spectrum

**Figure 2:**
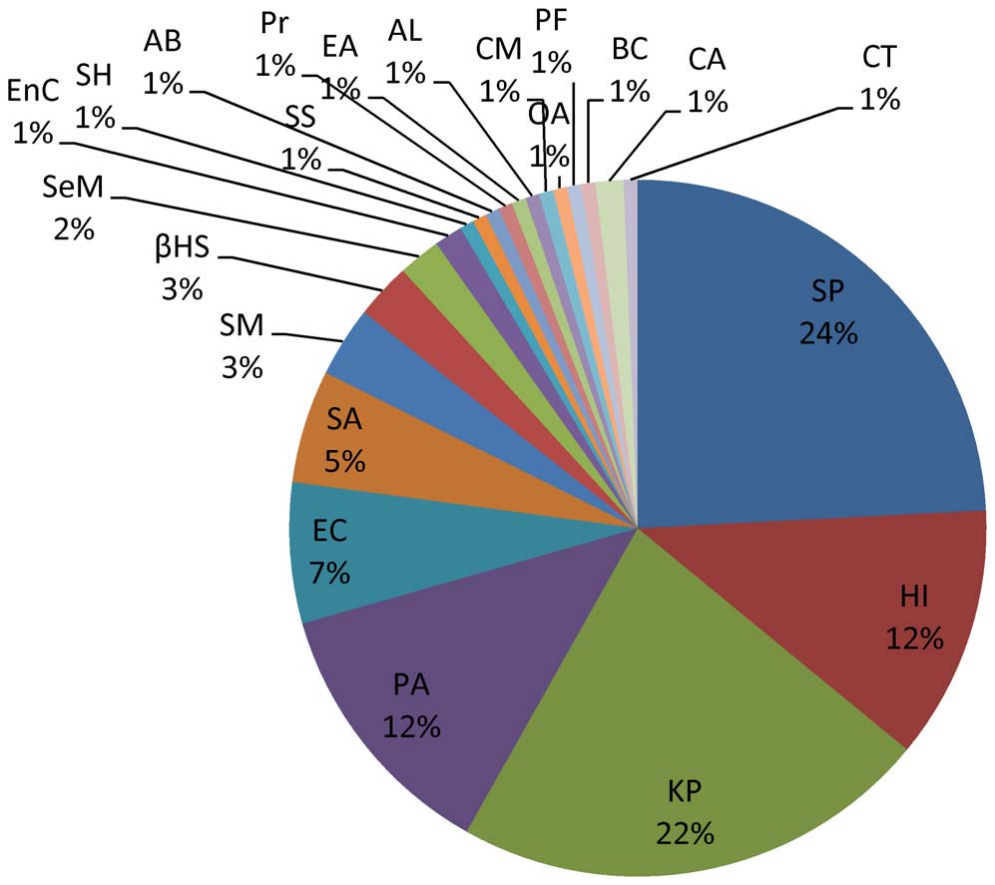
Etiological distribution of fever and respiratory syndrome in Guangxi, China, 2009–2011. Of 153 etiologies cultured from specimens of fever and respiratory syndromes: *Streptococcus pneumonia*(SP, 37 strains), *Haemophilus influenza*(HI,18), *Klebsiella pneumonia*(KP,34), *Pseudomonas aeruginosa*(PA,19), *Escherichia coli*(EC, 10), *Staphylococcus aureus*(SA,8), *Stenotrophomonas maltophilia*(SM,5), *β-hemolytic streptococcus*(βHS,4), *Serratia marcescens*(SeM,3), *Enterobacter cloacae*(EnC,2), *Staphylococcus hominis*(SH,1), *Staphylococcus saprophyticus*(SS,1), *Acinetobacter baumannii* (AB,1), Proteus(Pr,1), *Enterobacter aerogenes* (EA,1), *Acinetobacter lwoffii*(AL,1), *Chryseobacterium meningosepticum*(CM,1), *Ochrobactrum anthropic*(OA,1), *Pseudomonas fluorescens*(PF,1), *Burkholderia cepacia*(BC,1), *Candida albicans*(CA,2), *Candida tropicalis*(CT,1).

Of 153 (43.84%) etiologies cultured from specimens of fever and respiratory syndromes, pathogenic bacteria, opportunistic pathogens and fungus accounted for 35.95%, 62.09% and 1.96%, respectively. The most frequently isolated pathogens were *Streptococcus pneumonia* (37, 24.18%), followed by *Klebsiella pneumonia* (34 strains, 22.22%), *Pseudomonas aeruginosa* (19, 12.42%) and *Haemophilus influenza* (18, 11.76%) (Figure 2).

**Figure 3:**
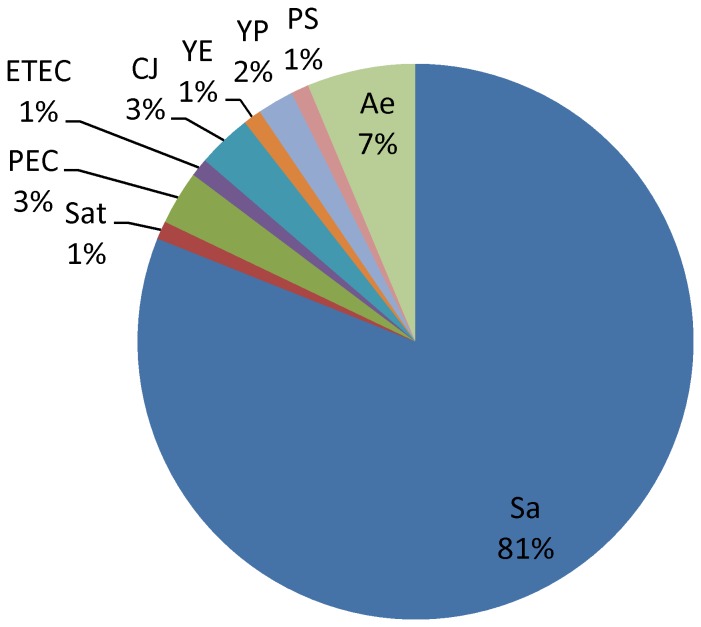
Etiological distribution of diarrhea syndrome in Guangxi, China, 2009–2011. Of 95 etiologies cultured from specimens of diarrhea syndromes: *Salmonella*(Sa,77), *Salmonella-typhi*(Sat,1), *Pathogenic Escherichia coli*(PE,3), *Enterotoxigenic Escherichia coli*(ETEC,1), *Campylobacter jejuni*(CJ,3), *Yersinia enterocolitica* (YE,1), *Yersinia pseudotuberculosis*(YP,2), *Plesiomonas shigelloides*(PS,1), *Aeromonas*(Ae,6).

Of 95 (27.22%) pathogens isolated from diarrhea syndrome specimens, pathogenic bacteria and opportunistic pathogens accounted for 90.53% and 9.47%, respectively. The most frequently isolated pathogens were *non-typhoidal Salmonella* (77, 81.05%), *Aeromonas* (6, 6.32%), *Pathogenic Escherichia coli* (6, 6.32%),and *Campylobacter jejuni* (3, 3.16%) (Figure 3).

**Figure 4:**
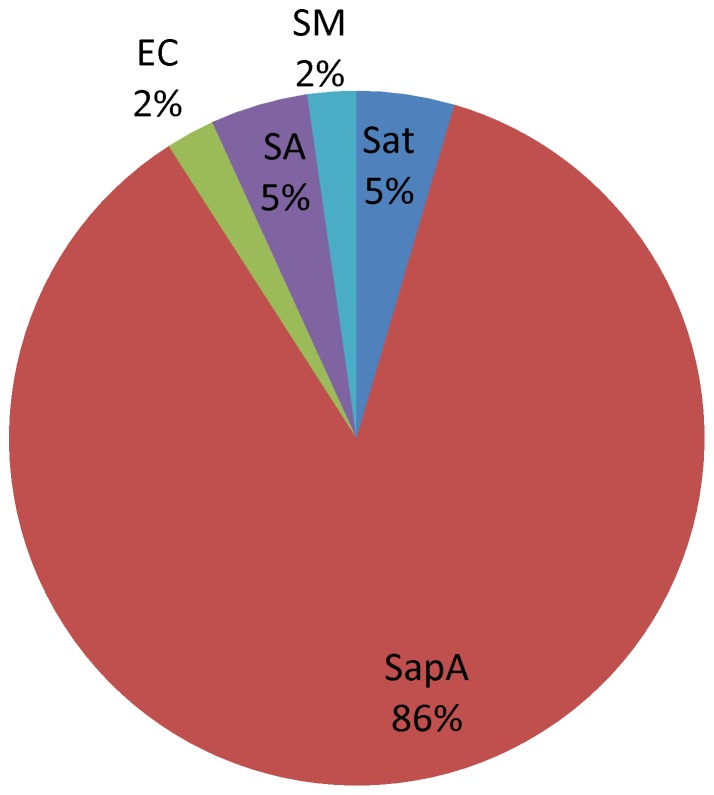
Etiological distribution of fever and rash syndrome in Guangxi, China, 2009–2011. Of 44 etiologies cultured from specimens of fever and rash syndromes: *Salmonella paratyphi A* (SapA,38 strains), *Salmonella-typhi* (Sat,2), *Escherichia coli*(EC,1), *Staphylococcus aureus* (SA,2), *Stenotrophomonas maltophilia*(SM,1).

Of 44 (12.61%) isolates cultured from specimens of fever and rash syndrome, pathogenic bacteria and opportunistic pathogens accounted for 93.18% and 4.55% of the isolates, respectively. The most frequently isolated pathogen was *Salmonella typhi A* (38, 86.36%), which was detected in cases caused by an outbreak of this syndrome (Figure 4).

**Figure 5:**
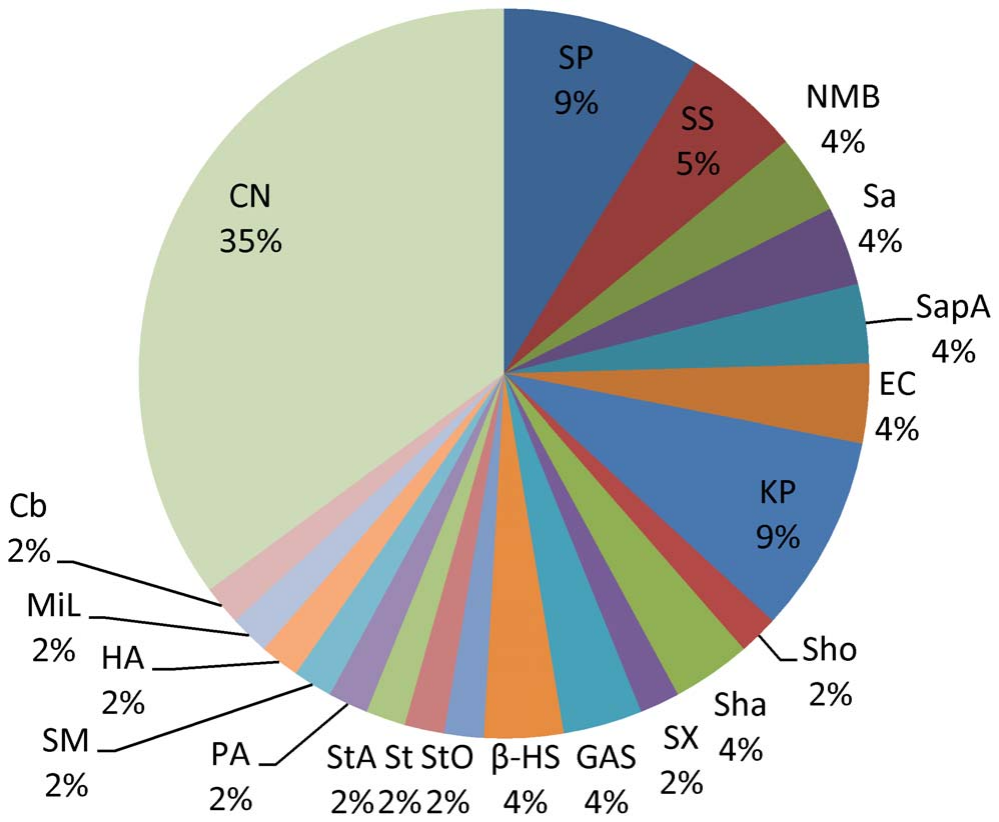
Etiological distribution of encephalitis-meningitis syndrome in Guangxi, China, 2009–2011. Of 57 etiologies cultured from specimens of encephalitis-meningitis syndromes: *Streptococcus pneumonia*(SP,5), *Streptococcus suis*(SS,3), *Neisseria meningitidis serogroup B*(NMB,2), *Salmonella*(Sa,2), *Salmonella paratyphi A*(SapA,2), *Escherichia coli* (EC,2), *Klebsiella pneumonia*(KP,5), *Staphylococcus hominis*(Sho,1), *Staphylococcus haemolyticus*(Sha, 2), *Staphylococcus xylosus*(SX ,1), *Group A streptococcus*(GAS,2), *β-hemolytic streptococcus* (β-HS, 2), *Streptococcus oralis*(StO, 1), *Streptococcus*(St,1), *Streptococcus agalactiae* (StA,1), *Pseudomonas aeruginosa* (PA,1), *Stenotrophomonas maltophilia*(SM,1), *Hafinia alvei*(HA,1), *Micrococcus luteus*(MiL,1), *Corynebacterium*(Cy,1), *Cryptococcus neoformans*(CN,20).

Of 57 (16.33%) isolates cultured from specimens of encephalitis-meningitis syndrome, pathogenic bacteria, opportunistic pathogens and fungus accounted for 31.58%, 33.33% and 35.09%, respectively. *Cryptococcus neoformans* was the most frequently isolated pathogen (20, 35.09%), followed by *Streptococcus pneumonia* (5, 8.77%), *Klebsiella pneumonia* (5, 8.77%),*Streptococcus suis* (3, 5.26%) and *Neisseria meningitides* group B (2, 3.51%) (Figure 5).

To date no bacterial pathogen has been detected from specimens of fever and hemorrhage syndrome.

### PFGE Analysis

**Figure 6:**
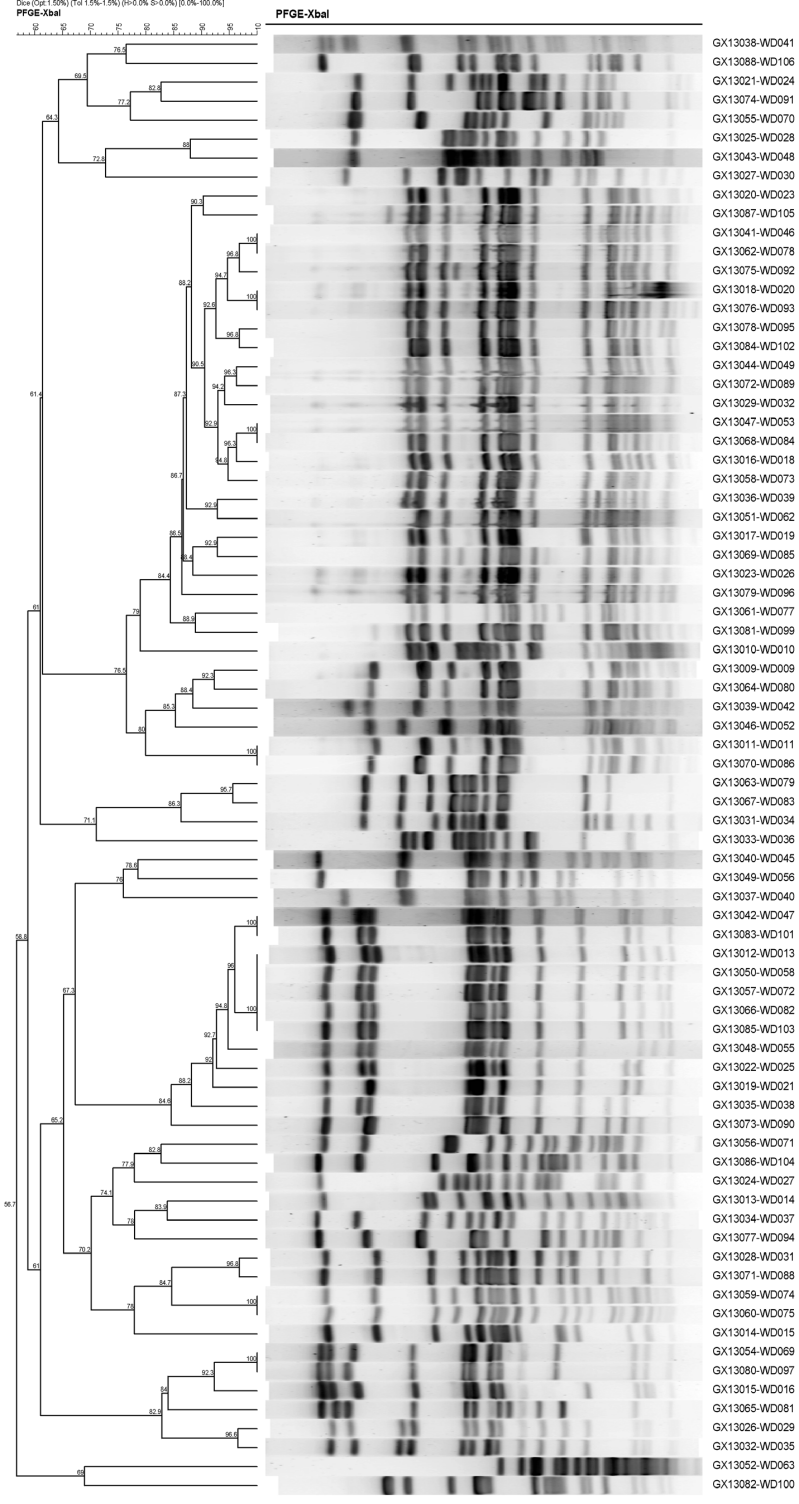
Comparison of PFGE patterns for isolates with high similarity.

A total of 112 strains of *Salmonella* were isolated in this surveillance system, making it the most commonly detected bacterial pathogen at present. PFGE assays indicated that these 112 *Salmonella* isolates were clustered in several pulsotypes. Figure 6 shows that identical PFGE patterns were observed in WD046 and WD078, WD020 and WD093, WD053 and WD084, WD011 and WD086, WD047 and WD101, (WD013, 058, 072, 082) and WD103, WD074 and WD075, WD069 and WD097.

Epidemiologic investigations conducted on those patients who had identical pulsotypes showed no obvious epidemiological links.

## Discussion

Under the present surveillance system, a total of 349 (5.91%) pathogen isolates were detected from 5,907 specimens collected from eligible cases of five core syndromes. In this study the pathogenic spectrum of five core syndromes in China was characterized for the first time.

The surveillance findings showed that the predominant pathogens attributing to fever and respiratory syndrome were *Klebsiella pneumonia, Haemophilus influenza* and *Staphylococcus aureus*, which was similar to previous reports from other countries [6]. Although vaccination against *Haemophilus influenzae* type b *(Hib)* has remarkably reduced the incidence of this disease in industrialized countries, the global burden of this disease remains high [7]. In China, *Hib* was one of the most important pathogens that caused pneumonia in children [8], although the disease burden of this pathogen is unclear and the currently reported incidence is believed to be an underestimate [9].

Under the surveillance system described here, *Salmonella* predominated in pathogens isolated from eligible cases of diarrhea syndrome. Previous surveillance conducted by various areas of China showed that *Salmonella* is one of the most important pathogens causing diarrhea [10], [11]. As such, more attention to the public health importance of *Salmonella* is needed due to its high infection rate and capacity to cause wide-spread infection. *Campylobacter jejuni* is a frequently reported pathogen that causes diarrhea worldwide. The findings of a twelve-year surveillance in Israel showed that the annual incidence rate of *C. jejuni* infection episodes increased from 24.59 to 70.54/100,000 cases during the period between 1999 and 2010 [12]. This organism is one of the most common causes of Guillain–Barré syndrome (GBS) [13], which may lead to severe signs and syndromes and can be life-threatening [14].

For encephalitis-meningitis syndrome, the most frequently detected organisms were *Cryptococcus neoformans*, *Streptococcus species* and *Staphylococcus species*, which was similar to findings obtained in Malawi [15]. However, a study in Italy showed that *Listeria monocytogenes* and *Neisseria meningitides* were the most common pathogens that caused meningitis in that country [16]. Meningitis caused by *C. neoformans* may lead to high morbidity and mortality [17] and is a common form of meningitis in immunocompromised patients [18] such as HIV/AIDS patients, as well as in long-term antimicrobial users. The HIV epidemic and increasing use of immunosuppressive therapies are believed to have contributed to the rising incidence of infections caused by *C. neoformans* [19]. Guangxi is one of the provinces in China with the most severe HIV epidemic and the most rapid increase in newly reported HIV-positive cases in recent years [20]. This may contribute to high proportion of *C. neoformans* detected in the study area.

*Streptococcus pneumoniae* is the most common cause of community-acquired pneumonia, meningitis and bacteremia in children and adults [21]. The incidence of pneumococcal diseases had significantly decreased in high-income countries as routine use of the pneumococcal conjugate vaccines [22]. Yet the high cost is an obstacle to introduction of these vaccines in resource-poor settings [23]. As a result, pneumococcal diseases remain important public health problems in low and middle income countries [24].

*Streptococcus suis* infections are a global problem in the swine industry [25] and can also cause severe invasive infection in humans who have close contact with infected pigs or contaminated pork [26]. In recent years, the number of reported *S. suis* infections in humans has increased significantly [27].

Following the introduction of the meningococcal vaccine, the incidence of meningococcal meningitis has been as low as 0.15/100,000 during the past decade in Guangxi. However, the case-fatality rate is still as high as 16.59% in this province. Under the surveillance system described here, serogroup B was isolated, which was rarely reported as compared to A and C during the past 10 years in Guangxi [28]. Serogroup B has now become the main causative agent in Europe and South America [29], since routine vaccination with serogroup C vaccines has drastically reduced the incidence of disease caused by this serogroup. In the absence of satisfactory vaccine against serogroup B meningococcal meningitis [30], a future epidemic outbreak caused by this serogroup could thus bring new challenges to disease control efforts in Guangxi and elsewhere.

In China, most previous investigations and laboratory testing on rash illness were aimed mainly at viral diseases [31], [32] and orientia tsutsugamushi [33], such that there was little information concerning other bacterial diseases. While the surveillance system described here attempted to reveal the bacterial pathogenic spectrum of fever and rash syndrome, limited bacterial pathogens (mainly *Salmonella paratyphi A*) were detected. As such, continuous surveillance will be needed to further explore the etiologic spectrum in Guangxi.

In this study no bacterial pathogen was detected from specimens of fever and hemorrhage syndrome. The infections causing hemorrhage syndrome were mainly reported in northern China rather than southern China and few cases of hemorrhage due to bacterial pathogens were previously reported by the routine surveillance system in Guangxi. This low incidence may be due to the low prevalence of these diseases or because testing for the bacterial pathogens was not routinely performed.

In addition to the common pathogenic bacteria found under the surveillance system described here, opportunistic pathogens have also drawn attention. Opportunistic pathogens have accounted for two-thirds of the pathogens for fever and respiratory syndrome, and one-third of meningitis syndrome. Echoing findings were noted in Kenya, where 35.29% of the bacterial pathogens were opportunistic pathogens in non-HIV patients with acute respiratory infection [34]. For meningitis, the proportion of opportunistic pathogens measured in Guangxi was higher than for Ghana (8.58%) [35]. A high proportion of opportunistic pathogens may be due to immunodeficiency [36], overuse of antimicrobials [37], increasing use of immunosuppressive therapies or nosocomial infection [38].

Under the present surveillance system, lower positive culture rates were found in sputum (14.71%), stool (10.08%) and blood (1.94%–10.55) specimens. The positive culture rate for sputum specimens was lower than the 27.5% [39] reported in Romania. For stool specimens, the rate in Guangxi was lower than that in Iran (45.6%) [40]. For blood specimens of respiratory infection patients, the positive culture rate was also lower when compared with findings from Thailand (9.2%) [41]. The positive rate (4.93%) for CSF culture in Guangxi was similar to that observed in Africa (3.3%–7%) [35], [42]. Antibiotic use outside hospitals without a medical prescription is very common in China and may affect the growth of bacteria in cultured specimens. The differences in positive rates might also be due to the changing pathogenic spectrum of infections. Previous research showed that the proportion of respiratory infection caused by bacteria has decreased, while the infection caused by viruses,mycoplasma and chlamydia has obviously increased [43], [44]. Viruses were also found to be the most important etiology of diarrhea [45].

In the surveillance system described here, *Salmonella* was selected for PFGE analysis, since for this pathogen sufficient numbers of strains have been collected to allow clustering analysis. Although a number of strains were found to have identical PFGE patterns, no epidemiological links were found between those patients infected with the identical strains. The strains with identical PFGE patterns were detected from patients living in different part of the city, by different sentinel hospitals and at different time points. There was no other case with similar syndromes reported for the patients enrolled in the same period. While no evidence showed that clustering of cases infected with *Salmonella* occurred, risk factors for infection still widely exist in this environment. Therefore, close monitoring is needed to avoid outbreaks caused by common factors (e.g. water or food contamination).

The surveillance system set up in Guangxi was an active syndromic surveillance based on laboratory findings that allowed the timely detection of bacterial infections with laboratory conformation. This system complements the national surveillance system, allowed the bacterial spectrum of infectious disease to be characterized, and infections caused by rare or newly emerging etiologies (or serogroups) to be identified. Moreover, implementation of this system allowed the disease control agencies to systematically monitor important pathogens which were not routinely detected in this area such as *Hib* and *Campylobacter jejuni* and to further study the etiologies using additional molecular biology assays. Implementation of such systems can also improve the detection sensitivity of pathogen such as *Streptococcus suis*, since cultures of this pathogen have been routinely performed on suspected cases of meningitis even without obtaining relevant epidemiologic information (e.g. frequent exposure to sick pigs or pork).

Implementation of an effective laboratory-based surveillance system requires close collaboration between the CDC system and the hospital system. Yet, it is still challenging to maintain a quality mechanism for public health surveillance due to insufficient understanding and lack of motivation from hospital staff. As such, strengthened cooperation between the CDC system and the hospital system, together with continuous training and education are needed to sustain the five-syndrome surveillance system in Guangxi.

## Limitations

The current surveillance system has not included viral pathogens and we can only obtain the bacterial spectrum without understanding the patterns of viral infection for the five core syndromes. Using the surveillance system described here, viral etiologies can be included to make the findings of the study more complete and valuable.

To date we can only characterize the PFGE patterns for *Salmonella* rather than other pathogens detected in our study. PFGE analysis has not been performed on those isolates because the number of the remaining strains is insufficient for performing clustering analysis. As the surveillance continues, we will conduct the PFGE analysis on the remaining pathogen isolates when sufficient number of strains is collected.
